# Polymer-based precipitation preserves biological activities of extracellular vesicles from an endometrial cell line

**DOI:** 10.1371/journal.pone.0186534

**Published:** 2017-10-12

**Authors:** Ziru Niu, Ronald T. K. Pang, Weimin Liu, Qian Li, Ranran Cheng, William S. B. Yeung

**Affiliations:** 1 Department of Obstetrics and Gynecology, Li Ka Shing Faculty of Medicine, The University of Hong Kong, Pokfulam, Hong Kong, China; 2 Center of Reproduction, Development, and Growth, Li Ka Shing Faculty of Medicine, The University of Hong Kong, Pokfulam, Hong Kong, China; 3 Shenzhen Key Laboratory of Fertility Regulation, The University of Hong Kong-Shenzhen Hospital; George Mason University, UNITED STATES

## Abstract

Extracellular vesicles (EVs) are membrane-bound vesicles released by cells and act as media for transfer of proteins, small RNAs and mRNAs to distant sites. They can be isolated by different methods. However, the biological activities of the purified EVs have seldom been studied. In this study, we compared the use of ultracentrifugation (UC), ultra-filtration (UF), polymer-based precipitation (PBP), and PBP with size-based purification (PBP+SP) for isolation of EVs from human endometrial cells and mouse uterine luminal fluid (ULF). Electron microscopy revealed that the diameters of the isolated EVs were similar among the tested methods. UF recovered the highest number of EVs followed by PBP, while UC and PBP+SP were significantly less efficient (P<0.05). Based on the number of EVs-to-protein ratios, PBP had the least protein contamination, significantly better than the other methods (P<0.05). All the isolated EVs expressed exosome-enriched proteins CD63, TSG101 and HSP70. Incubation of the trophoblast JEG-3 cells with an equal amount of the fluorescence-labelled EVs isolated by the studied methods showed that many of the PBP-EVs treated cells were fluorescence positive but only a few cells were labelled in the UC- and UF-EVs treated groups. Moreover, the PBP-EVs could transfer significantly more miRNA to the recipient cells than the other 3 methods (P<0.05). The PBP method could isolate EVs from mouse ULF; the diameter of the isolated EVs was 62±19 nm and expressed CD63, TSG101 and HSP70 proteins. In conclusion, PBP could best preserve the activities of the isolated EVs among the 4 methods studied and was able to isolate EVs from a small volume of sample. The simple setup and low equipment demands makes PBP the most suitable method for rapid EV assessment and isolation of EVs in clinical and basic research settings.

## Introduction

Extracellular vesicles (EVs) are nanometer-sized membrane-bound vesicles secreted by nearly all living cells into their extracellular environment. They are subcellular components enclosed in a membrane with a lipid bilayer. Exosomes, microvesicles and apoptotic bodies are different types of EVs. Exosomes are typically of sizes 30–100 nm in diameter [1]. They are formed within cells by inward budding of late endosomes, called multi-vesicular bodies. The exosomes are released into the extracellular environment when the multi-vesicular bodies fuse with the plasma membrane [2,3]. Different from the exosomes, microvesicles bud directly from the plasma membrane, and are usually 50–1000 nm in diameter. Apoptotic bodies have a wide range of sizes from 800 to 5000 nm [4–7]. Given the overlapping size ranges, it is well accepted that EV preparations are generally heterogeneous [8,9].

EVs represent an important mode of intercellular local (autocrine and paracrine) and remote communication transferring specific RNAs, proteins and lipids between cells [10,11]. It is increasingly clear that EVs mediate many diseases and physiological processes. The importance of EVs in the female reproductive tract and in mediating embryo-maternal cross talk is beginning to be recognized recently [12].

The most commonly used method for isolation of EVs is differential centrifugation, which involves multiple centrifugation and ultracentrifugation steps [4]. There are variations in the protocols of differential centrifugation with different recoveries of EVs [4]. Ultracentrifugation-free methods for isolation of EVs have also been developed, including ultra-filtration, precipitation and immuno-affinity capture using specific antibodies. Although studies have investigated factors affecting the efficiencies of these methods [13–16], there is no study comparing the biological activities of the EVs isolated by these methods.

A dialogue between endometrium and peri-implantation embryo is necessary for a successful implantation [17]. Some researchers hypothesize that EVs are involved in embryo-embryo communication and in embryo-endometrium cross talk [18]. EVs isolated from pregnant sheep uterine luminal fluid (ULF) transfer RNAs that can regulate trophectoderm development of conceptus [19]. EVs have been isolated from the ULF of women in different menstrual phases [12]. Both the luminal and glandular apical surfaces of endometrial epithelial cells express exosome enriched proteins, CD9 and CD63 [12], suggesting that the endometrial epithelium is a potential source of the exosomes in the ULF. Endometrial exosomes enhance the adhesive properties of human trophoblast cells [20]. However, the potential roles of EVs in embryo development is unclear [21].

In this study, we compared 4 EVs isolation methods. They were ultracentrifugation (UC), ultra-filtration (UF), precipitation (PBP), and precipitation with spin-column purification (PBP+SP). The pros and cons of these methods were studied before by others [14,16,22,23]. However, few of them compared the biological activities of the EVs isolated by these methods. Here, we focused on the biological activities of EVs isolated by these methods and their applicability in processing samples of small volumes. The latter is important as the availability of samples in implantation research is limited and often comes in small volume such as ULF.

## Material and methods

### Cell culture and preparation of conditioned culture medium (CCM)

Human endometrial adenocarcinoma cell line Ishikawa (ATCC, VA, USA) were cultured in 75 mm^2^ flasks with Minimum Essential Medium Eagle (MEM, Sigma-Aldrich, MI, USA) supplemented with 100 U/ml Penicillin-Streptomycin (P/S, Thermo Fisher Scientific, MA, USA), 1% L-glutamine (Thermo Fisher Scientific) and 10% fetal bovine serum (FBS, Thermo Fisher Scientific) at 37°C in an incubator with 5% carbon dioxide. EV-depleted-BSA was prepared by dissolving BSA in medium at a concentration of 5% followed by centrifugation at 100,000 g for 18 hours at 4°C. The resulting supernatant was collected and sterilized by passing through a 0.2 μm filter. For preparation of CCM, Ishikawa cells (~1×10^6^ cells) were seeded in a 75 mm^2^ flask, cultured for 24 hrs and washed three times with 5 ml PBS. The cells were then further cultured in 10 ml of MEM supplemented with P/S, L-glutamine and 1% EVs-depleted-BSA [4,24] for 48 hours. More than 95% of the cells remained viable after culture. The spent medium after 48 hours of culture was collected. Cell debris and large vesicles in the medium were removed by differential centrifugation. Briefly, the medium was centrifuged at 300 g for 10 mins to remove cells, 2,000 g for 10 mins to remove dead cells and 20,000 g for 60 mins to remove cell debris and large vesicles. After each centrifugation, the pellets were discarded and the supernatant was applied to the subsequent centrifugation [4]. The medium collected after the last centrifugation was designated as CCM.

### EVs prepared by ultracentrifugation (UC-EVs)

UC-EVs were isolated from CCM by ultracentrifugation (Hitachi Koki Himac CP100WX Ultracentrifuge, Tokyo, Japan) at 100,000 g in a fixed angle rotor (P90AT, Hitachi) for 80 mins at 4 ^o^C. The UC-EVs-pellet was resuspended in 200 μl of PBS. We and others [25] observed severe loss of EVs after washing, thus, the UC-EVs were not washed except in an experiment determining the effect of sample washing on EV internalization, in which the samples was washed as described [4]. The samples from this experiment were labelled as “UC+wash”.

### EVs prepared by ultra-filtration (UF-EVs)

UF-EVs were collected from CCM by centrifugation at 4,000 g for 20 mins through a Vivaspin 6 Centrifugal Concentrator (100k MWCO, Satorius, Goettingen, Germany). The UF-EVs was recovered from the stop pocket and topped up to 200 μl with PBS.

### EVs prepared by polymer-based precipitation (PBP-EVs)

PBP-EVs were isolated from CCM using the Total Exosome Isolation kit (Invitrogen, MA, USA) according to the manufacturer’s instructions. According to the manufacturer, the kit recovers mainly “exosomes” of sizes 50–150 nm, which is within the overlapping size ranges of exosomes and microvesicles. It is likely that the nanoparticles isolated contain both exosomes and microvesicles. Therefore, EVs instead of exosomes were used to describe the isolated nanoparticles [9]. Briefly, the total exosome isolation reagent was mixed with CCM in a ratio of 1:2 (v/v) and incubated overnight at 4°C on a roller mixer. Afterwards, the samples were centrifuged at 10,000 g for 60 mins at 4°C. The supernatant was discarded and the PBP-EVs pellet was resuspended in 200 μl of PBS. Unless stated, the PBP-EVs were not washed after centrifugation. To assess the effect of sample washing, 100 μl of PBS was used to wash the pellet before resuspension in 200 μl of PBS and the sample was labelled as “PBP+wash”.

### EVs prepared by PBP with size-based purification (PBP+SP-EVs)

PBP+SP-EVs were isolated from CCM using the Exo-spin™ Exosome Purification Kit (Cell Guidance Systems, Cambridge, UK) according to the manufacturer’s instructions. Briefly, CCM was mixed with Buffer A (2:1, v/v) and incubated overnight at 4°C on a roller mixer. Afterwards, the samples were centrifuged at 20,000 g for 60 mins at 4°C. The supernatant was discarded and the pellet was resuspended in 100 μl of PBS. The resuspension was loaded into a spin-column pre-washed with PBS. The loaded column was centrifuged at 50 g for 60 s. The fraction containing the PBP+SP-EVs was then eluted with 200 μl of PBS by low speed centrifugation for 1 min.

### Electronic microscopy (EM)

EVs were prepared for EM as previously described [4]. Briefly, EVs in PBS were fixed in equal volume of 4% (w/v) paraformaldehyde (PFA) and 1% (v/v) glutaraldehyde. The preparation was layered onto formvar-carbon coated 400 mesh copper grids (Ted Pella Inc, CA, USA) and dried at room temperature. The samples were then contrasted in a solution of uranyl oxalate (pH 7) and embedded in methyl cellulose-uranyl acetate (pH 4, 9 parts 2% methylcellulose and 1 part 4% uranyl acetate). The samples were examined under an electron microscope (Philips CM100 TEM, Eindhoven, Netherlands) with an accelerating voltage of 80 kV coupled to a camera (TENGRA 2.3K ×2.3k Olympus, Tokyo, Japan). The EM images were analyzed by the iTEM Acquisition Software (Olympus, Tokyo, Japan).

### SDS-PAGE and Western blotting

EVs were lysed, denatured in SDS sample buffer (0.4% SDS, 0.2M Tris-HCl, pH 6.8, 5% glycerol, 0.02% bromophenol blue, 1% betamercaptoethanol) at 95°C for 10 mins and resolved in 10% SDS PAGE. Details of western blotting procedures were described elsewhere [26]. Expression of TSG101 (sc-7964, Santa Cruz TX, USA), CD63 (ab8219, Abcam, Cambridge, UK) and HSP-70 (YM3042, ImmunoWay. TX, USA) were detected by specific antibodies. The quantities of protein in the EV preparations were determined by the Pierce BCA Protein Assay kit (Thermo Fisher Scientific) according to the manufacturer’s instructions. EV-to-protein ratio was determined by the quotient of the number of EVs/ml and the protein concentration.

### Quantitation of EVs

The EXOCET Exosome Quantitation Assay Kit (SBI, CA, USA) was used to quantify the amount of EVs according to the manufacturer’s instructions [27,28]. It is based on determination of acetyl-CoA acetylcholinesterase (AChE) activity, which is enriched in exosomes. Briefly, total protein concentration of the preparations was determined by the Pierce BCA Protein Assay kit as described above. EVs (50 μg) were lyzed with lysis buffer provided by the manufacturer and incubated at 37°C for 5 minutes to release the proteins. Standard curve of the assay was prepared from the enzyme standard available from the manufacturer. The enzyme activities of the samples and the standards were determined by incubation in a reaction buffer in 96-well plates at room temperature for 20 mins. The optical density was measured at 406 nm by a spectrophotometric plate reader (Infinite 200, TECAN, Männedorf, Switzerland).

### Quantitative RT-PCR of mature miRNAs in EVs

After EVs isolation, the quantities of EVs isolated by different methods were normalized by the expression levels of HSP70 determined in Western blotting. EV preparations were mixed with water in a ratio of 4:1 (v/v). The mixture was incubated at 95°C for 10 mins to lyse the EVs and to release the vesicular RNAs. The quality of the RNAs was determined by a NANODROP 2000 (Thermo Fisher Scientific). Quantitative RT-PCR was carried out using TaqMan microRNA assays (Applied Biosystems, CA, USA) according to the manufacturer’s instructions. Reverse transcriptions for miRNA was performed as described [26] with a 7500 Real Time PCR System (Applied Biosystems). All the reactions, including no-template controls (RNase-free deionized water), were run in triplicate. After the reactions, the CT values were determined using default threshold settings. TRIzol reagent (Thermo Fisher Scientific) was used to isolate cellular total RNA according to the manufacturer’s instructions. Quantitative assessment of relative changes of miRNAs expressions in cells was calculated by the comparative CT method (2^-△△CT^) [26]. △CT was used to show the differences between target miRNAs and internal reference (small nucleolar RNA, RNU6). The amounts of target miRNAs in EVs were expressed as CT values [29].

### Internalization of EVs into trophoblast cells and mouse blastocysts

The fluorescent membrane dye FM1-43FX (Invitrogen) at a final concentration of 5 μg/ml was added to the CCM to label the EVs by incubation at room temperature for 5 mins. The CCM was then subjected to the EV isolation procedures described above. After EVs isolation, the quantities of EVs isolated were normalized by the expression levels of HSP70. JEG-3 cells, a human placenta choriocarcinoma cell line (ATCC, VA, USA), was used as the EVs recipient trophoblast cells. The cells were co-cultured with the labeled EVs in medium containing 5% EVs-depleted-BSA for 24 hrs at 37°C. Afterwards, the cells were washed twice with PBS, fixed with 4% PFA at 4°C for 15 mins and then washed again with PBS. The nucleus of the cells was visualized by staining with DAPI at a concentration of 1 μg/ml in PBS (Invitrogen) at room temperature for 20 mins and the cytoskeleton was stained by Phalloidin (Cell signaling technology, MA, USA) at room temperature for 15 mins. The cells were washed with PBS thrice before fluorescence microscopy (Carl Zeiss LSM 700 confocal system, ZEISS, Jena, Germany). The images were analyzed by the ZEN software (ZEN 2010 version 6.0.0.309, CO, USA).

For the blastocyst EVs internalization assay, blastocysts were collected from Day 4 pregnant imprinted-coding region (ICR) mice (6–8 weeks, 34.5±3.5 g). Three mice were used for each experimental group and each experiment was repeated three times. The protocol was approved by the Committee on the Use of Live Animals in Teaching and Research, The University of Hong Kong (CULATR number: 3560–15). ICR female mice were superovulated as previously described [30]. The mice were euthanasia by overdose of pentobarbital (150–200 mg/kg, i.p.). Preimplantation embryos at Day 4 developmental stages were retrieved by flushing the uterus of the pregnant ICR females with PBS. The labeled EVs were cocultured with the embryos in KOSM+AA medium (Millipore, MA, USA) at 37°C for 24 hours. For better visualization, the cytoplasm of blastocysts was labelled with Qtracker Cell labeling kit (Invitrogen) or Phalloidin. Briefly, the labeling solution was prepared according to the manufacturer’s instructions and was added to 20 μl of KOSM+AA medium. Labeling was performed for 60 mins. The blastocysts were washed with PBS for 3 times before fixation.

### The ability to transfer miRNA to recipient cells

Let-7a-enriched EVs were prepared by transfection of pre-let-7a (Ambion, CA, USA) into Ishikawa cells in 6-well culture plate as described [26]. Pre-miR scramble was used as a control. After transfection, the transfection medium was replaced with fresh MEM medium supplemented with 1% P/S, 1% L-glutamine and 5% EVs-depleted-BSA. The CCM derived from the transfected cells was collected and subjected to various EVs isolation methods. The let-7a enriched EVs were co-cultured with the recipient JEG-3 cells in DMEM/F12 supplemented with 5% EVs-depleted-BSA for 24 hrs before the let-7a expression in the JEG-3 cells was measured. Lin28a is a target of let-7a [31]. After the co-culture of let-7a enriched EVs with JEG-3 cells, the lin28a (Cell signaling technology, Cat no. 3978) expression were detected by fluorescence microscopy. The images were analyzed by the ZEN software.

### Inhibition of exosomes production

GW4869 is a pharmacological chemical for blocking of exosome generation [32]. The Ishikawa cells were treated with GW4869 at 0.1 μM, 1 μM, 10 μM or 20 μM in 5% EVs-depleted-BSA for 48 hours and the EVs were isolated by PBP and UC. Same number of cells was used for different treatments. The effects of the treatment on cell viability and EVs production were assessed by proliferation assay (Invitrogen, Cat no. C35006) and XTT assay (Roche, Cat no.11465015001), the expression of HSP70 and EVs quantitation in the treated cells, respectively.

Cambinol is another inhibitor of neutral sphingomyelinase 2, which blocks exosome production [33]. Similar to GW4869, the Ishikawa cells were treated with cambinol at concentrations of 0.1 μM, 1 μM, 10 μM or 30 μM in 5% EVs-depleted-BSA for 48 hours and the EVs were isolated by PBP. Same number of cells was used for different treatments. The effect was assessed as the above.

### EVs isolation from mouse uterine luminal fluid (ULF) by PBP

PBP method was applied to isolate EVs in mouse ULF. Briefly, Day 4 pregnant ICR mice (3 mice for each experiment group and each experiment was repeated three times) were sacrificed by overdose of pentobarbital (150–200 mg/kg, i.p.) and their uteri were isolated. ULF was collected by flushing the uterine lumen of 3 mice with 500 μl of PBS, which was then subjected to PBP isolation. The EVs were characterized by EM and expression of specific EV-enriched proteins by Western blotting.

### Statistical analysis

All images of Western blotting, EM and confocal microscopy were representatives of at least three independent experiments. Quantitative RT-PCR assays were performed in triplicate, and each experiment was repeated for at least five times. Data shown were presented as means ±SD. Differences were considered statistically significant at p < 0.05 using one way ANOVA as appropriate by SPSS 13.0 (IBM, NY, USA).

## Results

### Ultrastructural characterization of isolated EVs

**Fig 1:**
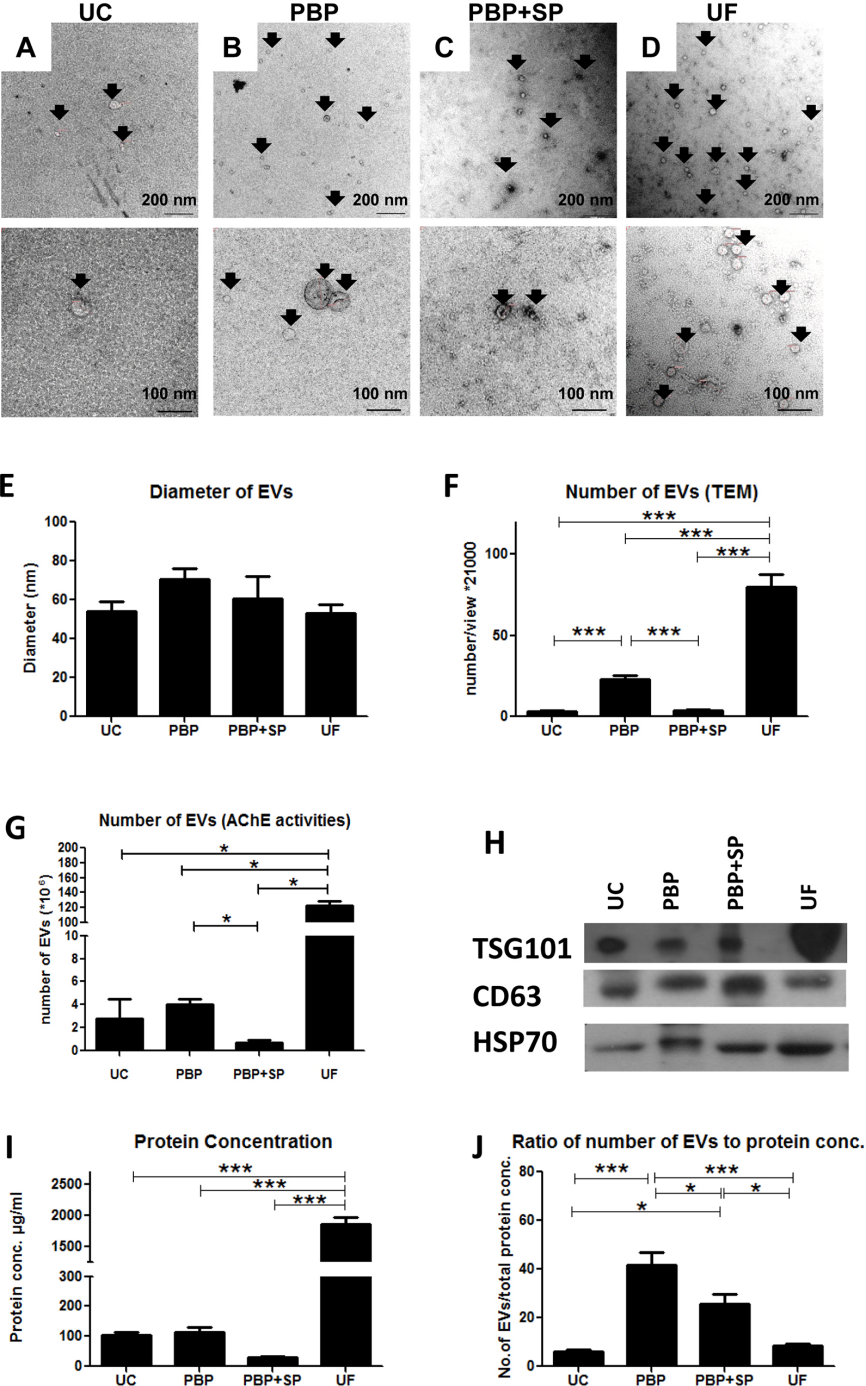
Properties of the isolated EVs. Ultrastructure of EVs isolated by UC **(A)**, PBP **(B)**, PBP+SP **(C)** and UF **(D)** under electron microscope with magnification of x21,000 (upper panel) and x52,000 (lower panel). **(E)** Diameter of EVs determined by electron microscopy. **(F)** Number of EVs counted/view under electron microscope. **(G)** Number of EVs measured by acetyl-CoA acetylcholinesterase (AchE) activity. **(H)** Western blotting showing expression of exosome-enriched proteins TSG101, CD63 and HSP70 in the preparations. **(I)** Protein concentration of the EV preparations. **(J)** Ratio of number of EVs/protein concentration.

The ultrastructure of the EVs derived from Ishikawa cells was analyzed by EM (Fig 1A–1D). All the tested methods generated a heterogeneous mixture of membrane-bound vesicles. The morphology and size of the isolated vesicles were consistent with previous report [34]. There was no significant difference in size between the EVs isolated by the 4 different methods (Fig 1E, UC: 54.04±17.03 nm, PBP: 70.71±35.16 nm, PBP+SP: 60.43±45.40 nm and UF: 53.30±27.58 nm).

The recoveries of EVs by the 4 methods were compared by determining the mean number of EVs per view for a total of 60 different fields under EM at a magnification of 21,000 times. The data revealed that UF recovered the highest number of EVs, followed by PBP, and UC was the least efficient in recovering EVs (Fig 1F, Mean number of EVs/field: PBP: 23.0±9.0, PBP+SP: 3.5±2.5, UF: 79.9±25.0 and UC: 3.2±2.6). Besides, the amount of EVs was also estimated by their acetyl-CoA acetylcholinesterase activity. The results were expressed as mean number of EVs and showed a pattern similar to that obtained by EM, i.e. UF has the highest recovery while PBP+SP has the least number of EVs (Fig 1G: PBP: 4.0±0.9, PBP+SP: 0.7±0.5, UF: 122.9±9.0 and UC: 2.8±3.3). Since this method is based on enzyme activities of the EVs, it is a rough estimation of the number of EVs. On the other hand, the EM method employs a direct counting of the number of EVs and is a standard method in EV quantification [35]. Therefore, the results of EV counting were used to determine the amount of EVs used in subsequent experiments.

### Protein expressions of the isolated EVs

There is no universal loading control for Western blotting of EVs. It is common to use total protein loading as internal control for EV Western blotting [36,37]. The same amount of protein of each preparation was loaded for Western blotting. The results showed expression of exosome-enriched proteins CD63, TSG101 and HSP70 in all the EVs preparations (Fig 1H). Hence BCA protein assay showed that the UF preparation carried a lot of proteins and the protein concentration was significantly higher than all other preparations (Fig 1I). It is known that proteins from cells or culture medium can contaminate EV preparations [25,38,39]. The purity of EV preparations was presented as quotient of mean number of EVs/field and protein concentration. As EM counting is a gold standard in EV quantification [35], we used the number of EVs obtained from EM for calculating the EV-to-protein ratio. A high EV-to-protein ratio indicated low protein contamination and vice versa. PBP had a EV-to-protein ratio significantly higher than the other preparations (Fig 1J, PBP:41.67±8.77, PBP+SP: 25.78±6.76, UF: 8.66±0.86 and UC: 6.21±1.16, * p<0.05).

### Small RNA expression in the EVs preparations

**Fig 2:**
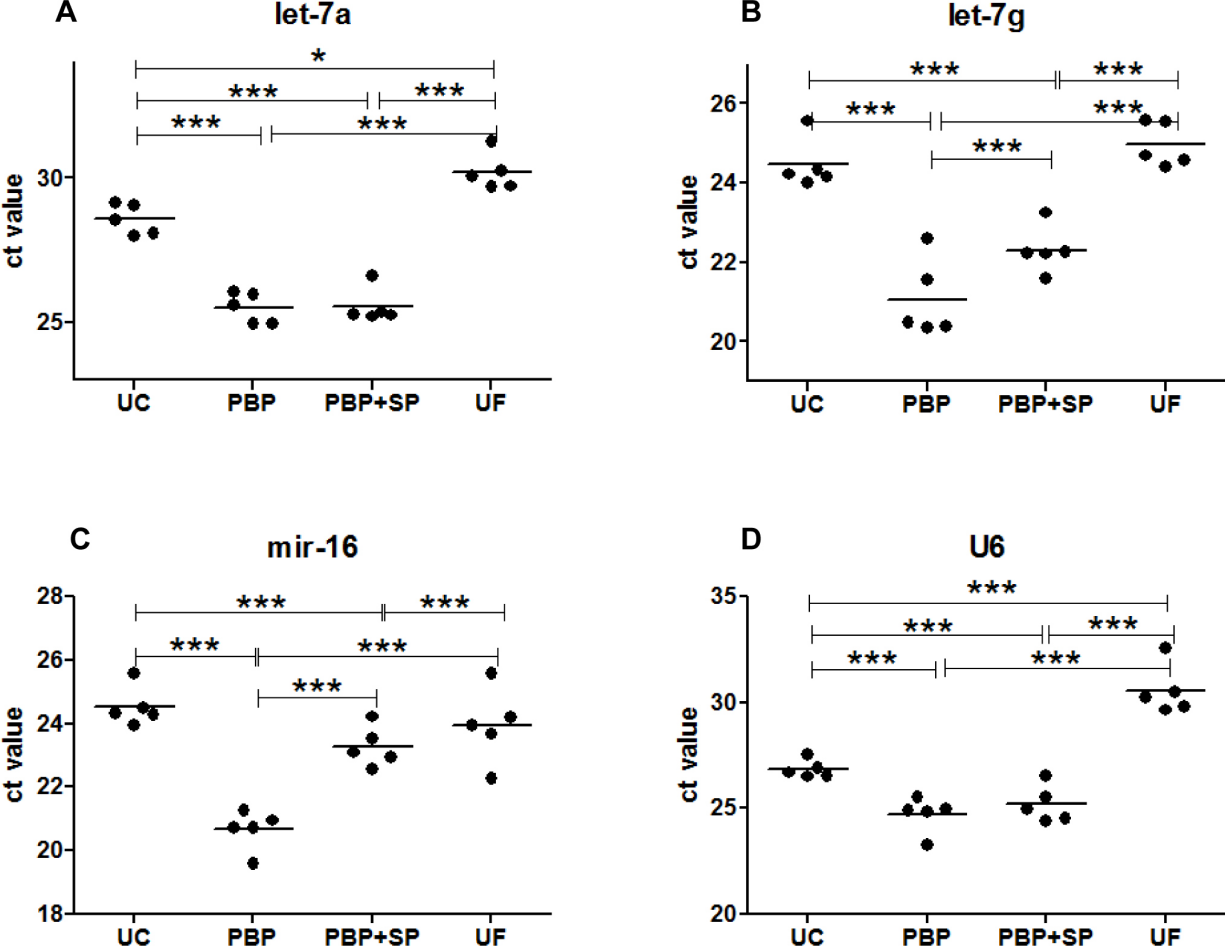
Expression of miRNA/mRNA in the EVs preparation. **(A)** Let-7a, **(B)** Let-7g, **(C)** miR-16 and **(D)** U6. Expression levels are presented as Ct values of quantitative PCR. Each circle indicates the Ct values of the captioned miRNA. Each experiment was repeated five times. The loadings for quantitative PCR were normalized by expression of HSP70 protein in the Western blotting.

The miRNA expressions in the EVs preparations were detected by miRNA TaqMan assays (Fig 2A–2D). Here, the miRNA expressions were presented by Ct values and were normalized by the level of HSP70 starting materials. The Ct values of miRNAs Let-7a (UC:28.4±0.6, PBP:25.7±0.6, PBP+SP: 25.3±0.1 and UF: 29.8±0.2), Let-7g (UC:24.2±0.2, PBP:20.4±0.1, PBP+SP: 22.0±0.4 and UF: 24.6±0.1), U6 (UC:26.6±0.1, PBP:24.9±0.1, PBP+SP: 24.6±0.3 and UF: 30.0±0.5), miR-16 (UC:24.4±0.1, PBP:20.8±0.1, PBP+SP: 22.9±0.3 and UF: 23.9±0.3) were significantly lower in the PBP preparation than in other preparations. A higher Ct value means lower expression. Therefore, the expression of let-7a and let-7g was highest for the PBP-EVs and the PBP+SP-EVs. The Ct values in PBP preparation were also lower than other preparations if the isolated EVs were reconstituted to the same volume (S1 Fig). We observed that U6B was only occasionally detected in some of the preparations.

### Internalization of labeled EVs into trophoblast cells and mouse blastocysts

**Fig 3:**
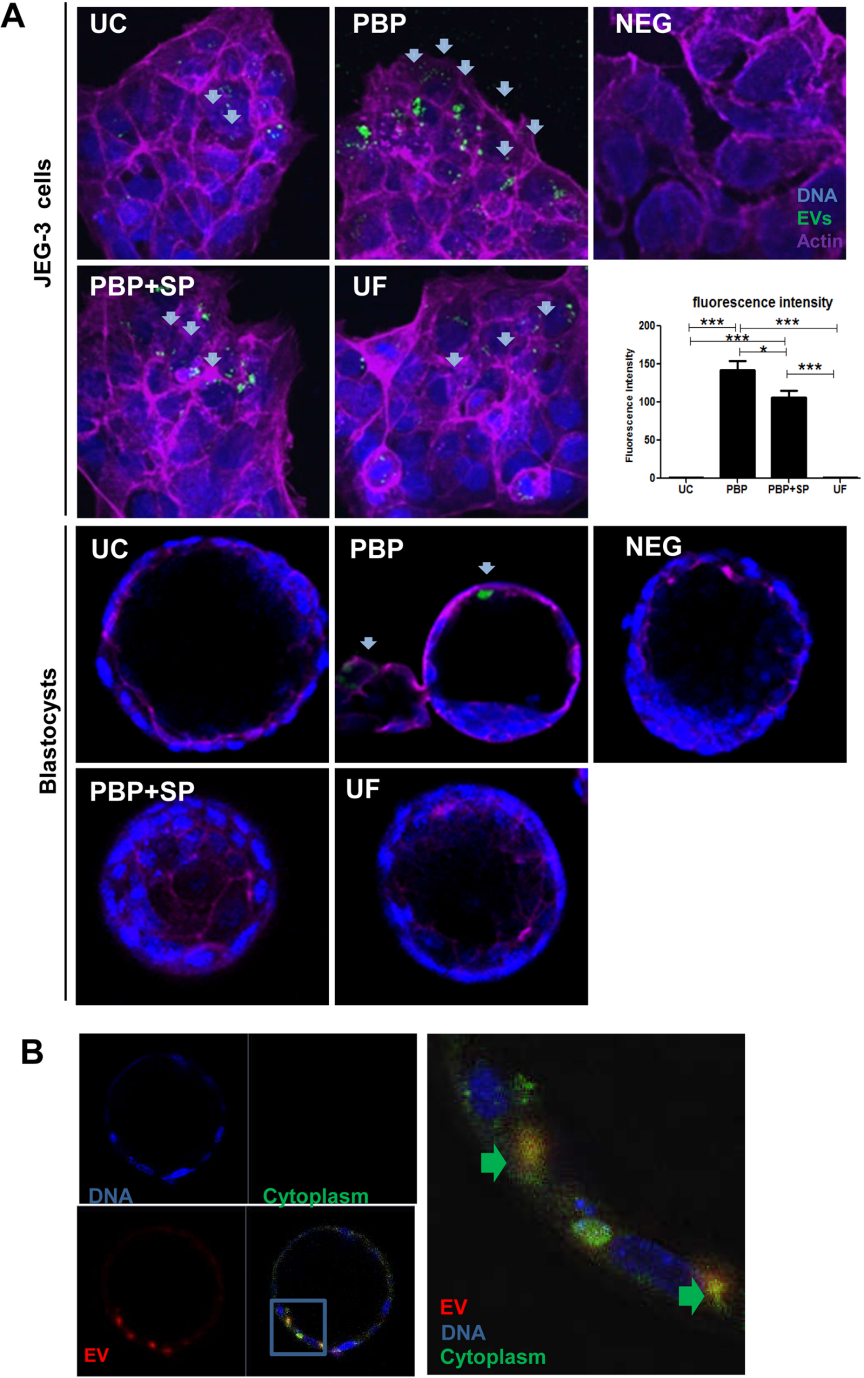
Internalization of EVs into JEG-3 cells and mouse blastocysts. **(A)** Confocal microscopy showing internalization of labelled EVs to JEG-3 cells (upper panel) and blastocysts (lower panel). Arrows indicates labelled EVs inside cells. Bar chart compares the fluorescence intensity of the labelled cells. **(B)** Magnified view of labelled EVs in trophectoderm cells. The cytoplasm of trophectoderm cells were labelled with Qtracker (Green). Magnified view (right) clearly shows that the EVs are present in the cytoplasm of the trophectoderm. Arrows indicate EV internalized into the cytoplasm.

EVs in the CCM of Ishikawa cells were isolated by the tested methods and were fluorescently-labeled. Same number of labeled EVs from different preparations was cocultured with JEG-3 cells or mouse blastocysts. Confocal microscopy revealed that the JEG-3 cells cocultured with PBP-EVs had the highest number of fluorescently-labeled cells while only a few cells were labeled with use of the UC- or UF-EVs preparations (Fig 3A, upper panel). The florescence intensity of the JEG-3 cells that had been cocultured with PBP-EVs was significantly higher than that of the other groups (PBP:141.86±24.97, PBP+SP: 105.74±19.65, UC:1.01±0.01, and UF: 1.00±0.01 intensity unit). To determine whether the higher internalization efficiency of PBP-EVs is due to the effect of residual PBP reagent, a control experiment was performed, in which the same amount of PBP reagent (1:20 in volume) was added to UC-EVs. We found that added PBP reagent did not affect internalization of EVs into Jeg3 cells (S2 Fig). Besides, an additional washing step was performed on EVs isolated by UC and PBP precipitation. Confocal microscopy showed that removal of possible contamination by washing did not affect the efficiencies of EV uptake (S3 Fig).

Similar results were obtained when blastocysts were cocultured with labeled EVs; more labeled cells were observed in blastocysts cocultured with the PBP-EVs preparation than those with the other preparations (Fig 3A, lower panel). To confirm that the PBP-EVs were internalized into the blastocysts, the blastocysts were labeled with Q-tracker, a cytoplasm specific dye. Confocal microscopy revealed that the labeled EVs (red) were located in the cytoplasm of the trophectoderm cells (green, Fig 3B).

### Transfer of EVs miRNA into recipient trophoblast cells

**Fig 4:**
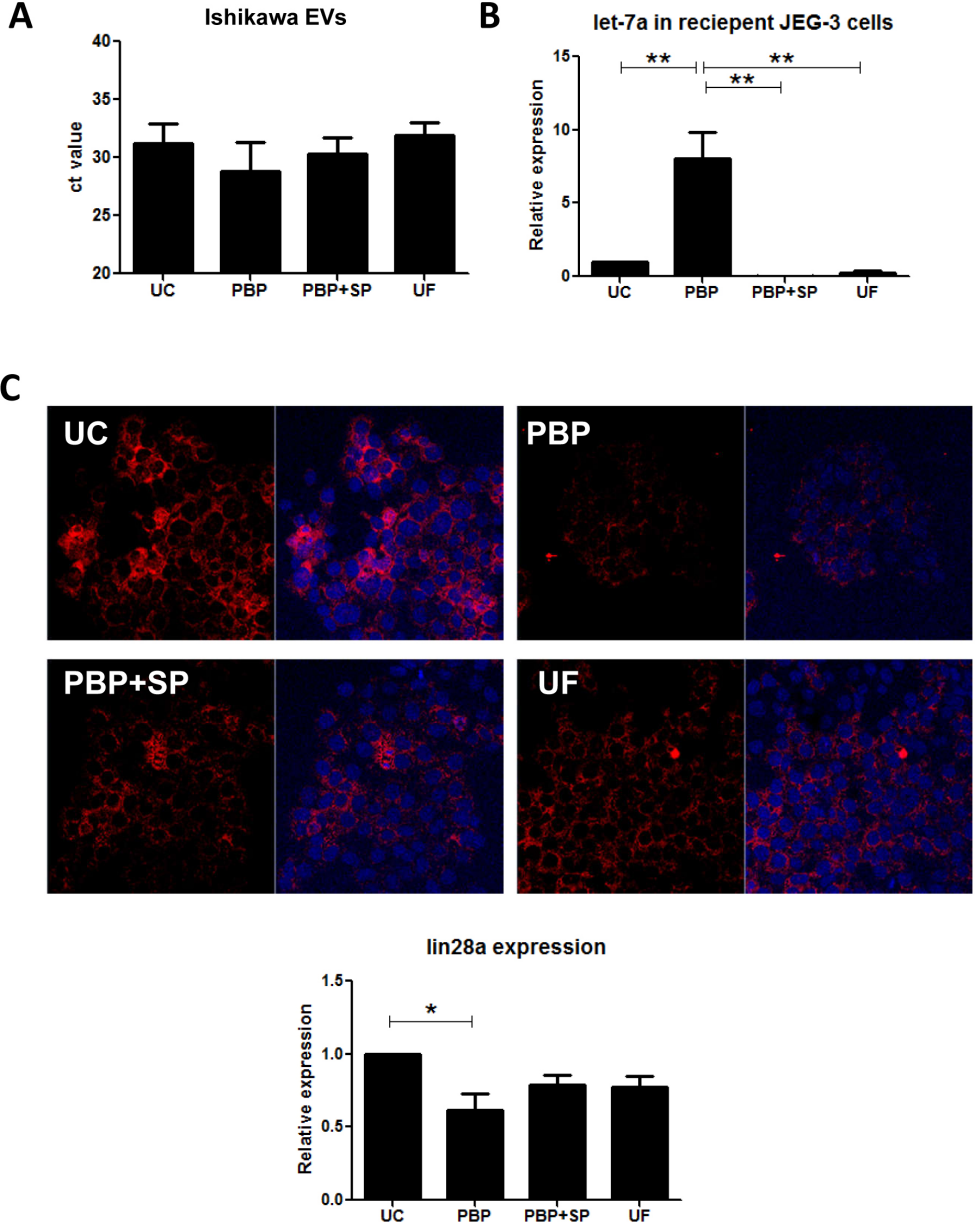
Internalization of EVs miRNA to recipient trophoblast cells. **(A)** Let-7a expression in the EVs isolated from Ishikawa cells overexpressing let-7a. **(B)** Expression of let-7a in the recipient JEG-3 cells cocultured with EVs isolated by different methods. Lower panel shows that lin-28a was significantly suppressed in the PBP group when compared to UC.

To study whether the EVs from various isolation methods had different miRNA transfer ability, Ishikawa cells were transfected with let-7a, a miRNA associated with embryo dormancy [40], and their EVs were isolated by the 4 studied methods. Equal number of the let-7a-enriched EVs was used to co-culture with the JEG-3 trophoblast cells. The let-7a expression in the EVs and the recipient JEG-3 cells were measured (Fig 4A). The expression of let-7a in the isolated EVs was higher in the PBP-EVs (CT value: 28.8±6.22) than those EVs isolated by other methods (CT value: PBP+SP: 30.36±4.17, UC: 31.24±4.77 and UF: 31.87±3.37). Besides, PBP-EVs are more efficient in transferring let-7a into the recipient JEG-3 cells when compared to the UC, UF and PBP+SP EVs (PBP 8.06±3.04-fold, PBP+SP: 0.016±0.01-fold, UC:1.0±0-fold and UF: 0.25±0.21-fold, Fig 4B). Confocal microscopy confirmed suppression of Lin28a expression in the JEG-3 cells (Fig 4C) in the PBP-EVs when compared with UC-EVs. The results indicated that the PBP-EVs could transfer miRNAs into the recipient trophoblast, inhibiting the target genes expression.

### GW4869 and Cambinol significantly reduced the yield of PBP-EVs

**Fig 5:**
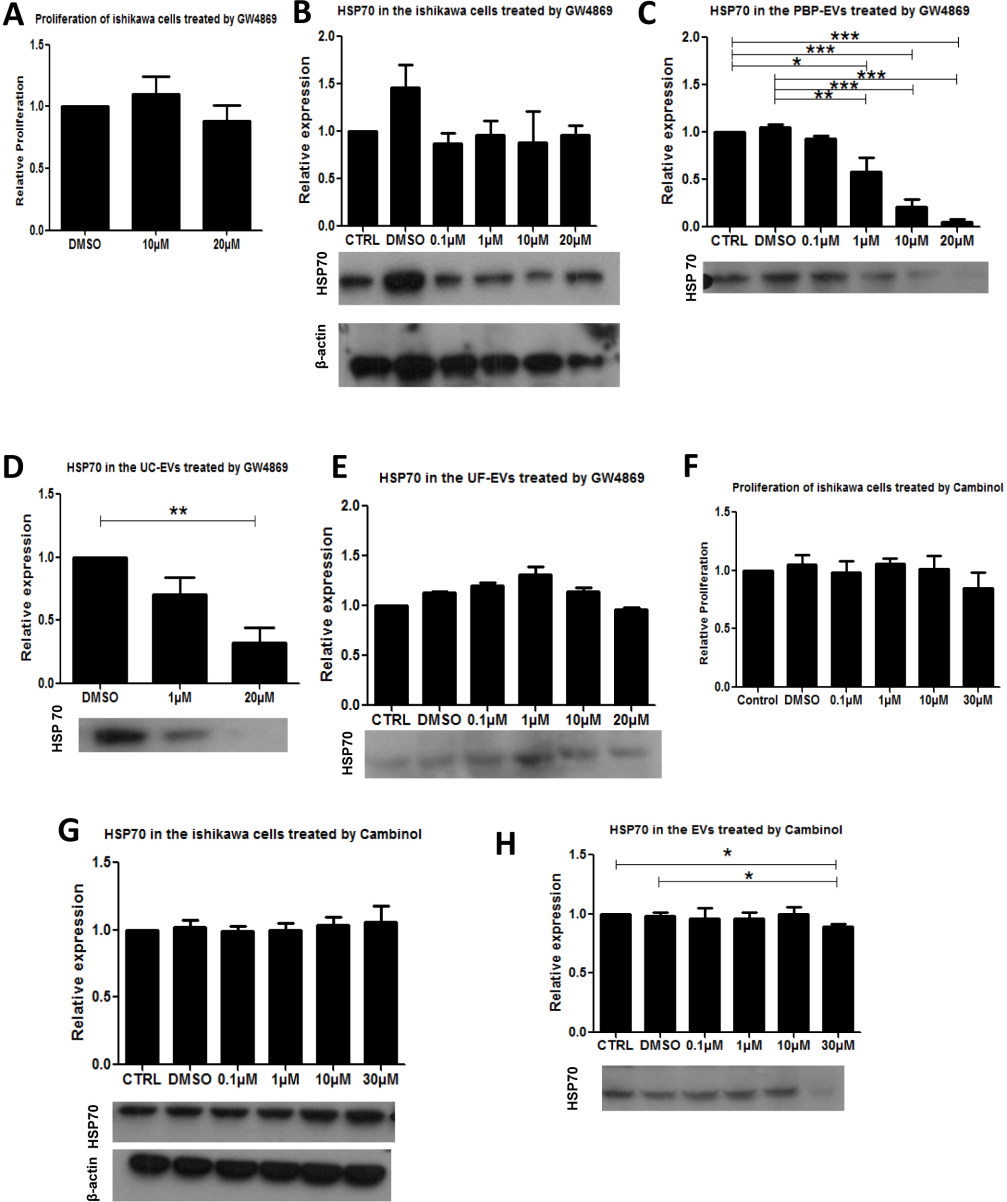
Verification of PBP in EVs isolation. GW4869 treatment does not affect proliferation **(A)** and relative HSP70 expression **(B)** of Ishikawa cells. GW4869 inhibits the amount of PBPEVs in the Ishikawa cells as reflected by a reduction of HSP70 in the PBP-EV preparations **(C)**. UC preparation show similar trend as PBP preparation **(D)**. Cambinol treatment does not affect proliferation **(E)** and relative HSP70 expression **(F)** of Ishikawa cells. **(G)** Cambinol inhibits the quantity of PBP-EVs in the Ishikawa cells as reflected by a reduction of HSP70 in the PBP-EV preparations.

To verify that the detected exosome-enriched protein in the PBP preparation was indeed associated with EVs, we treated Ishikawa cells with GW4869, an inhibitor of exosome biogenesis/release. Treatment with the inhibitor at concentrations that did not affect cell proliferation and intracellular expression of HSP70 (Fig 5A and 5B and S4 Fig) significantly and dose-dependently reduced HSP70 in the PBP-EVs preparation (Fig 5C) consistent with a reduction of the total EVs isolated. GW4869 also significantly reduced HSP70 in the UC-EVs preparation (Fig 5D). The effect of Cambinol, another inhibitor of exosome production, was also studied. The inhibitor at concentration from 0.1 μM to 30 μM did not affect cell proliferation and intracellular expression of HSP70 (Fig 5E and 5F). However, at 30 μM, it significantly reduced the amount of EVs production (Fig 5G).

### PBP could isolate EVs from mouse ULF

**Fig 6:**
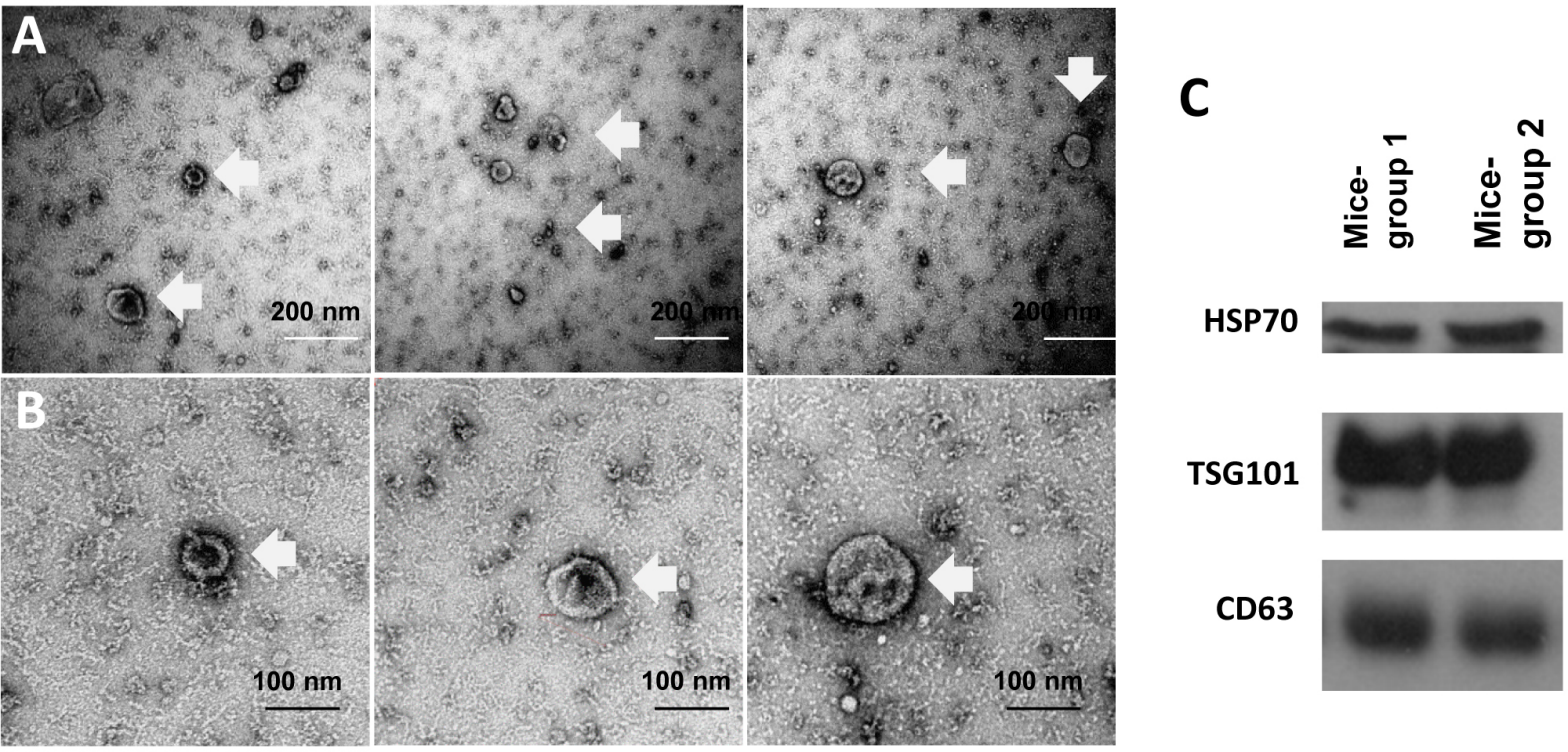
Characterization of EVs isolated by PBP from mouse ULF. Ultrastructure of mouse ULF-EVs **(A)** 21,000x magnification **(B)** 52,000x magnification. **(C)** Presence of HSP70, TSG101 and CD63 markers in the mouse ULF-EVs.

To examine whether the PBP method could be applied to EVs isolation from small volume of samples, it was used to isolate the EVs from mouse ULF. Electron microscopy showed that the EVs isolated from the mouse ULF had the expected morphology of EVs with a mean diameter of 61.66 ± 19.4 nm (Fig 6A and 6B). Western blotting showed that the EVs contained CD63, TSG101 and HSP70 (Fig 6C).

## Discussion

An ideal isolation method of EVs should 1) isolate EVs with high purity efficiently; 2) preserve the biological activities of the isolated EVs; 3) be capable of scaling up or down easily; 4) require minimal manpower and skills; and 5) not require expensive equipment. In our hands, we showed that PBP fulfilled the above criteria, and was better than other isolation methods tested in this study. Although there are studies on biological activities of EVs isolated by various methodologies, we and others [41] are not aware of studies comparing the biological effects of EVs isolated by different EV isolation methodologies.

UC was the original method for purifying EVs and was once the “gold standard” method for exosome preparation [42]. It involves multiple centrifugation steps that remove cells and cellular debris, followed by an ultracentrifugation step (>100,000 g centrifugal force) for pelleting exosomes. In some reports, the initial low speed centrifugation for cell/debris removal was replaced by a size exclusion filtration [43,44]. Other EVs isolation techniques like antibody-coated immunobeads [45], ELISA plates [46], UF [47], affinity chromatography [48] and PBP [49] have been developed recently. While these techniques have been used by many researchers, the biological activities of the isolated EVs by these methods remain unclear. Recently, Rekker and coworkers [50] compared UC and three commercially available reagents for isolation of serum exosomes. MiRNA quantitative PCR demonstrated that the commercial kits have performance similar to that of UC.

In our hands, all the tested methods isolated EVs of similar size, morphology and expression of exosomal enriched proteins. The advantages and disadvantages of these methods are summarized in Table 1. UC is the most widely used method for EVs isolation [51,52]. Our results showed that the recovery of UC-EVs was poor, and that the UC-EVs had reduced ability of being internalized or transferring miRNA. These observations suggested that the UC-EVs had reduced biological activities. The protein concentration of the UC preparation was similar to that of the PBP preparation, but the number of EVs in the preparation was seven times less than that of the PBP preparation, indicating that the former was contaminated by other proteins. Other studies also showed that UC did not solely sediment the vesicles but also some non-vesicular materials [53]. Some UC preparation could be heavily contaminated with proteins to an extent that interfered identification of exosomal proteins [25,38,39]. The type, quantity and quality of the vesicles isolated by UC is sensitive to multiple parameters; including g-force, rotor type (fixed angle or swinging bucket), angle of rotor sedimentation, radius of the centrifuge, pelleting efficiency (rotor and tube k-factors), and solution viscosity. Controlling all these parameters at the same time is difficult [54]. The applicability of UC in clinical practice is limited because it is time-consuming and cannot process a large number of samples simultaneously.

**Table 1:** The advantages and disadvantages of different EV isolation methods.

Methods	Advantages	Disadvantages
**Ultracentrifugation** **(UC)**	• Most commonly used method; • No additional reagent required.	• Requires special equipment; • Time consuming; • Cannot process a large number of samples simultaneously; • Damages isolated vesicles, reducing their quality.
**Ultra Filtration (UF)**	• Quick and simple; • Process many samples simultaneously; • No additional reagent required.	• Protein contamination. • Poor biological activities.
**Precipitation (PBP)**	• Simple and easy; • High yield of EVs; • Easy for subsequent RNA profiling; • Process many samples simultaneously.	• High reagent cost.
**Precipitation + size purification (PBP+SP)**	• High purity; • Process many samples simultaneously.	• Low yield; • Reduced bioactivities.

EVs can be separated from soluble proteins and aggregates using UF columns containing matrices with defined size exclusion limits. This is a quick and simple isolation method without the need for high centrifugal force or special chemicals. Some researchers believe that the UF method is more suitable for clinical use than the UC method [55]. Here, UF isolated the highest number of EVs among the methods tested. However, the low number of EV-to-protein ratio indicated that there was significant protein contamination in the preparation, possibly due to blockage of the UF nanomembrane and thereby reduction in efficiency of removing protein contaminants [56]. The number of UF-EVs isolated was around 3.5-times higher than that of the PBP-EVs but the protein concentration was around 17-times higher. Unexpectedly, UF-EVs did not exhibit the highest biological activities. They also had lower miRNA content in general. These observations reflected that UF might cause damage to the EVs by an unknown mechanism leading to reduction in biological activities and/or leakage of contents. In theory, EVs should only be found in the upper pocket of the UF columns, though we noticed the presence of a lot of EVs in the filtrate under EM (data not shown).

The principle of PBP is to use a reagent that binds to water molecules and thereby forcing the less-soluble components, such as EVs, to sediment out of the solution, allowing them to be collected by a short, relatively low-speed centrifugation. Among the tested methods, PBP yielded the second highest numbers of EVs with the highest EV-to-protein ratio. According to our miRNA analysis, the mean miRNA Ct values were significant lower in the PBP-EVs than in EVs collected by other methods, indicating that the total miRNA in EVs isolated from an equal amount of CCM was highest in the PBP preparation. Taylor and coworkers compared the efficacy of different extraction methods and showed that PBP resulted in a higher yield of miRNA with greater purity than chromatography, UC and DynaBeads techniques [49].

More importantly, PBP is the best method to preserve the bioactivities of EVs among the methods tested in our study. PBP-EVs are more easily internalized and therefore, they can transfer more miRNAs into the recipient cells. Taken together, The PBP method is simple, fast and requires only a standard low speed centrifuge. It yields EVs of good quantity and quality, can be adapted for use with small amount of starting material, and is suitable for downstream molecular analyses and simultaneous processing of multiple samples.

Apart from EVs, PBP also precipitates proteins [14] and lipoproteins [50]. In line with a previous study, we showed that the protein contamination of PBP is less severe than that of UC [57]. A disadvantage of PBP is the high cost of the reagents, which is around 4 USD/ml of sample. However, if ultracentrifuge is not readily available, the PBP method is a convenient alternative without the need for expensive equipment. Compare with UC, PBP has a shorter learning curve, and is less labor intensive making it more convenient to fit into routine laboratory. As a washing step was not included in the manufacturer’s protocol, users should pay attention to the effect of contaminating materials that may affect experimental outcomes.

In order to remove the contaminating proteins, a size exclusion chromatographic step, such as use of Exo-spin columns, is added to PBP aiming to separate the proteins from the EVs based on their size differences. Thus the PBP+SP preparations have less protein contamination [6,58], consistent with our observation. However, the higher purity was accompanied by a loss of EVs, and that forcing EVs through a size exclusion column may deform and rupture the EVs [53]. It has been suggested that the size exclusion step should be performed by gravity or with use of the lowest centrifugal force [53,59]. Moreover, the selection of the appropriate gel type is crucial to the recovery of EVs [41].

In this study, it is unlikely that the protein contamination in the EV preparations have significant effect on our conclusion in the internalization experiments. The EVs were prepared from 48-hr spent cell culture media. Vast majority of the contaminants in the EVs preparations should be derived from the BSA supplement in the culture media. The medium used for the internalization experiment also contained BSA and therefore the same components as in the contaminants. These components in the fresh medium are present in much larger quantity than that in the added EV preparations, and would mask any possible effect due to different amount of the contaminants among EV preparations.

Due to resources constrain, we were unable to test all the published methods and protocols. Further studies are needed to compare the ability of the untested methods in preserving the biological activities of EVs. Studies have demonstrated that longer spin time and higher centrifugal force can improve isolation efficiency of UC [54] but it has been suggested that the shear stress of prolonged centrifugation may affect the integrity of EVs [60,61]. Prolonged centrifugation may also induce aggregation of EVs [62] and increase soluble protein contamination [54]. Immunoaffinity isolation is another commonly used methods, it captures EVs on beads based on expression of specific surface markers of EVs such as CD63 and CD81. However, it may only isolate subpopulations of marker-positive EVs and there is doubt on functionality of the captured EVs after elution from the capture beads [4]. Besides, the method is not suitable for samples with large volumes [4].

EVs are actively released by cancer cells into the peripheral circulation [48] and researches on using exosomal proteins and RNA profiles as biomarkers in cancer diagnosis are ongoing [63,64]. Similar concepts are now being applied to determine endometrial receptivity. It is proposed that endometrial cells release EVs with contents that reflect the receptive status of the endometrium. EVs in ULF can be collected easily in a noninvasive manner either by aspiration or lavage prior to embryo transfer in an assisted reproduction treatment cycle. We found that PBP could isolate EVs from small volume of mouse ULF, and that ULF-EVs have size similar to that of human EVs and express exosomal enriched proteins.

GW4869 is more effective than Cambinol in inhibiting EV production. In fact, previous studies showed that the IC_50_ of GW4869 and Cambinol are 1 and 5 μM respectively [33,65]. Thus, at the same concentration, GW4869 is more effective than Cambinol. Cambinol at concentration higher than 30 μM reduced proliferation of Ishikawa cells, thus, the experiments were performed with a dose 30 μM of Cambinol and at this concentration production of EV was significantly suppressed although to a lesser extent than GW4869.

A limitation of our study is that we only used Ishikawa cell EVs as a model to demonstrate the effectiveness of the different isolation methods. In addition, it is known that the complexity of the fluids from which EVs are to be isolated affects the efficiency of the isolation and composition of the non-EV contaminants [66]. Spent chemically defined culture media used in this study is a relatively simple fluid. Therefore, modification of the current protocol is likely to be required for isolation of EVs from in vivo body fluid.

In summary, compared with the methods tested, PBP has the advantages of being simple, fast and effective for the isolation of EVs. It also preserves the bioactivity of the isolated EVs, and can be applied for isolation of EVs from tiny volume of biological fluid. Our findings indicated that biological activity and miRNA delivery efficiency of EVs could be affected by different isolation methods.

## Supporting information

S1 Fig Expression of miRNA/mRNA in the EVs preparation which is normalized by the starting volume. **(A)** Let-7a, **(B)** Let-7g, **(C)** miR-16, **(D)** U6 and **(E)** U6B. Expression levels are presented as Ct values of quantitative PCR. Each circle indicates the Ct values of the captioned miRNA. Each experiment was repeated five times. The loadings for quantitative PCR were normalized by starting isolation volume of each sample.
(JPG)

S2 Fig Internalization of EVs was not enhanced by PBP reagent. Internalization of UC-EVs into JEG-3 cells with or without PBP reagent was compared. The percentages of EV-positive cells were similar between the 2 groups indicated that PBP reagent did not enhance EV internalization.
(JPG)

S3 Fig Internalization of EVs were not affected by an additional washing step. Left panel: Confocal microscopy showing internalization of labelled EVs into JEG-3 cells. Right panel: Bar chart and table showing percentage of EV-labeled JEG-3 cells. The uptakes of UC-EVs and UC-washed-EVs in JEG-3 cells were similar. Similar results were also observed in the PBP-EVs and PBP-washed-EVs group. There was no significant difference between samples with or without additional washing step of the same isolation method. Sample washing did not affect internalization of PBP-EVs.
(JPG)

S4 Fig The effect of GW4869 and Cambinol on Ishikawa cells were assessed by XTT assay. All the tested doses and the scramble control were not significantly different.
(JPG)
