# Supplementary figures and images for: Polymer-based precipitation preserves biological activities of extracellular vesicles from an endometrial cell line
Source: PLoS One. 2017 Oct 12;12(10):e0186534. doi: 10.1371/journal.pone.0186534 (PMC5638560; doi:10.1371/journal.pone.0186534)

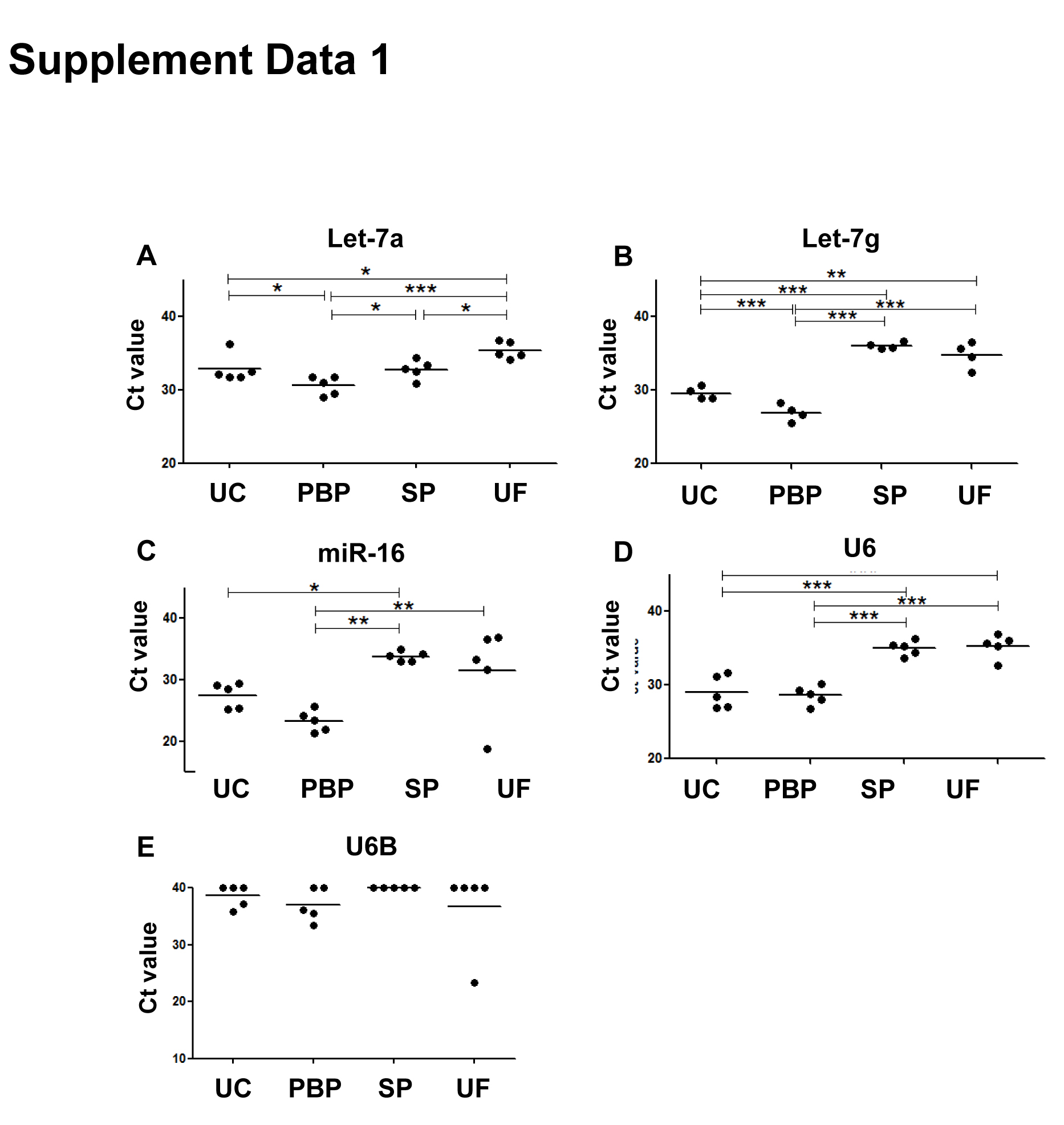

Supplement: S1 Fig — (A) Let-7a, (B) Let-7g, (C) miR-16, (D) U6 and (E) U6B. Expression levels are presented as Ct values of quantitative PCR. Each circle indicates the Ct values of the captioned miRNA. Each experiment was repeated five times. The loadings for quantitative PCR were normalized by starting isolation volume of each sample. (JPG) [file pone.0186534.s001.jpg]

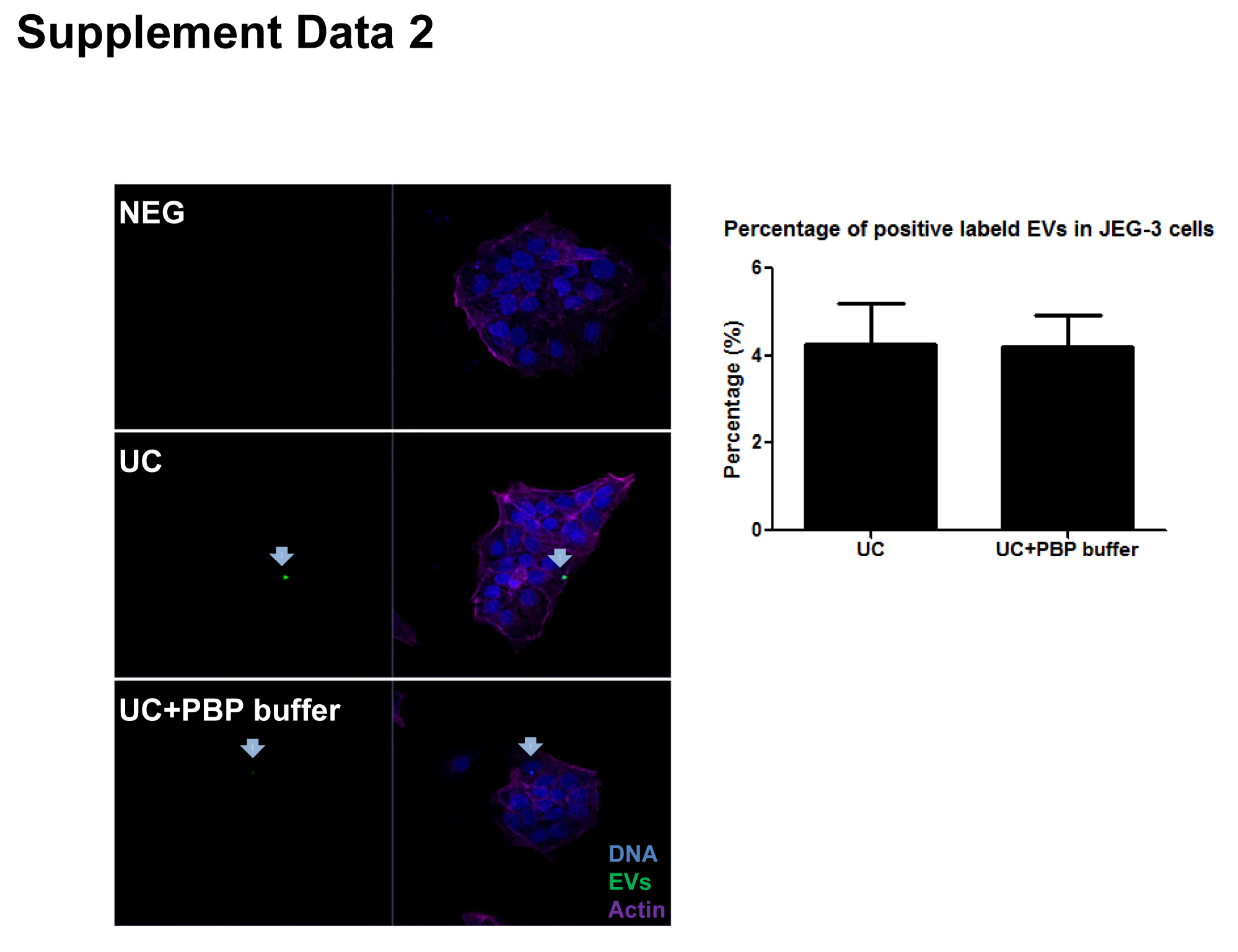

Supplement: S2 Fig — Internalization of UC-EVs into JEG-3 cells with or without PBP reagent was compared. The percentages of EV-positive cells were similar between the 2 groups indicated that PBP reagent did not enhance EV internalization. (JPG) [file pone.0186534.s002.jpg]

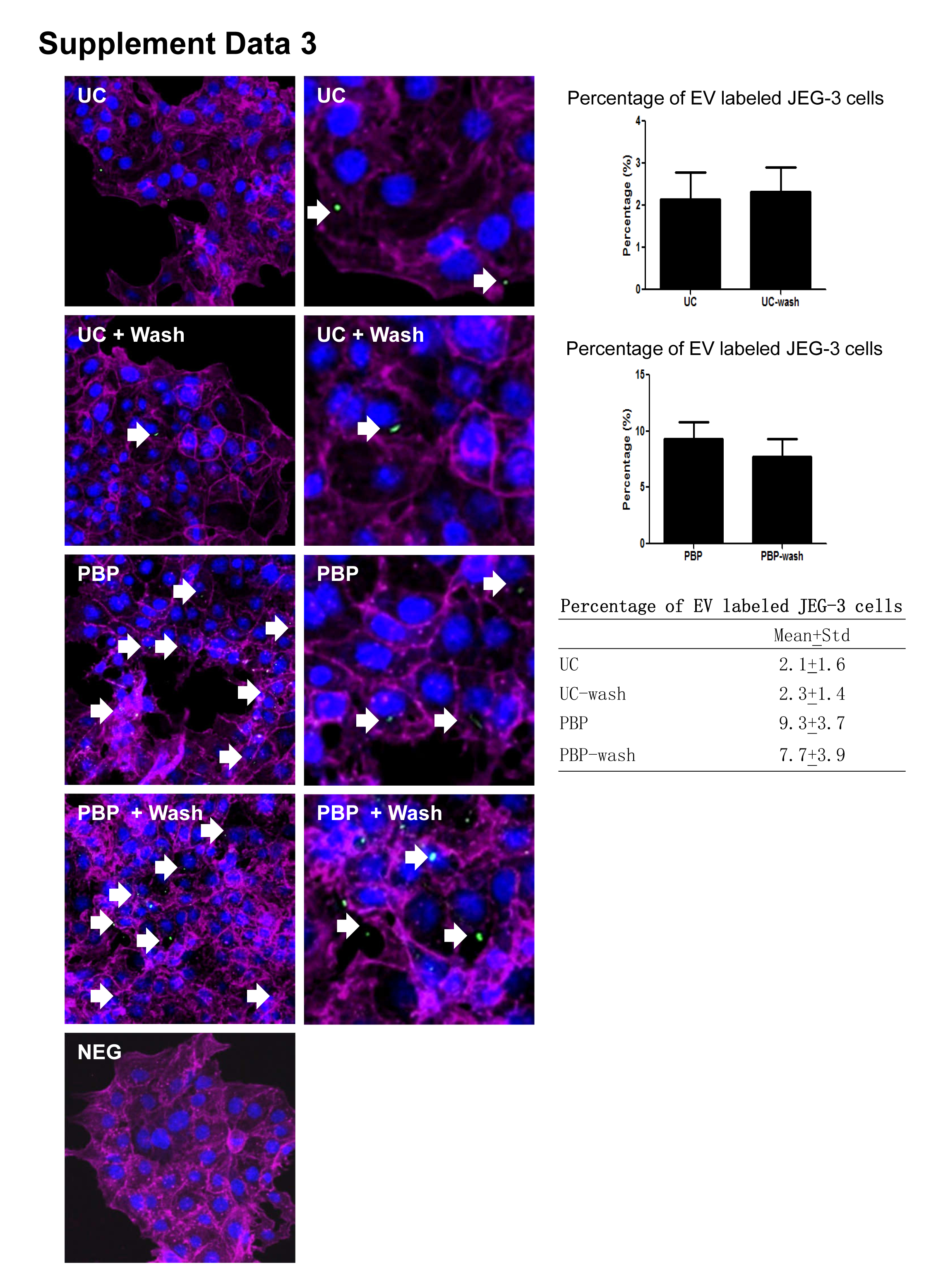

Supplement: S3 Fig — Left panel: Confocal microscopy showing internalization of labelled EVs into JEG-3 cells. Right panel: Bar chart and table showing percentage of EV-labeled JEG-3 cells. The uptakes of UC-EVs and UC-washed-EVs in JEG-3 cells were similar. Similar results were also observed in the PBP-EVs and PBP-washed-EVs group. There was no significant difference between samples with or without additional washing step of the same isolation method. Sample washing did not affect internalization of PBP-EVs. (JPG) [file pone.0186534.s003.jpg]

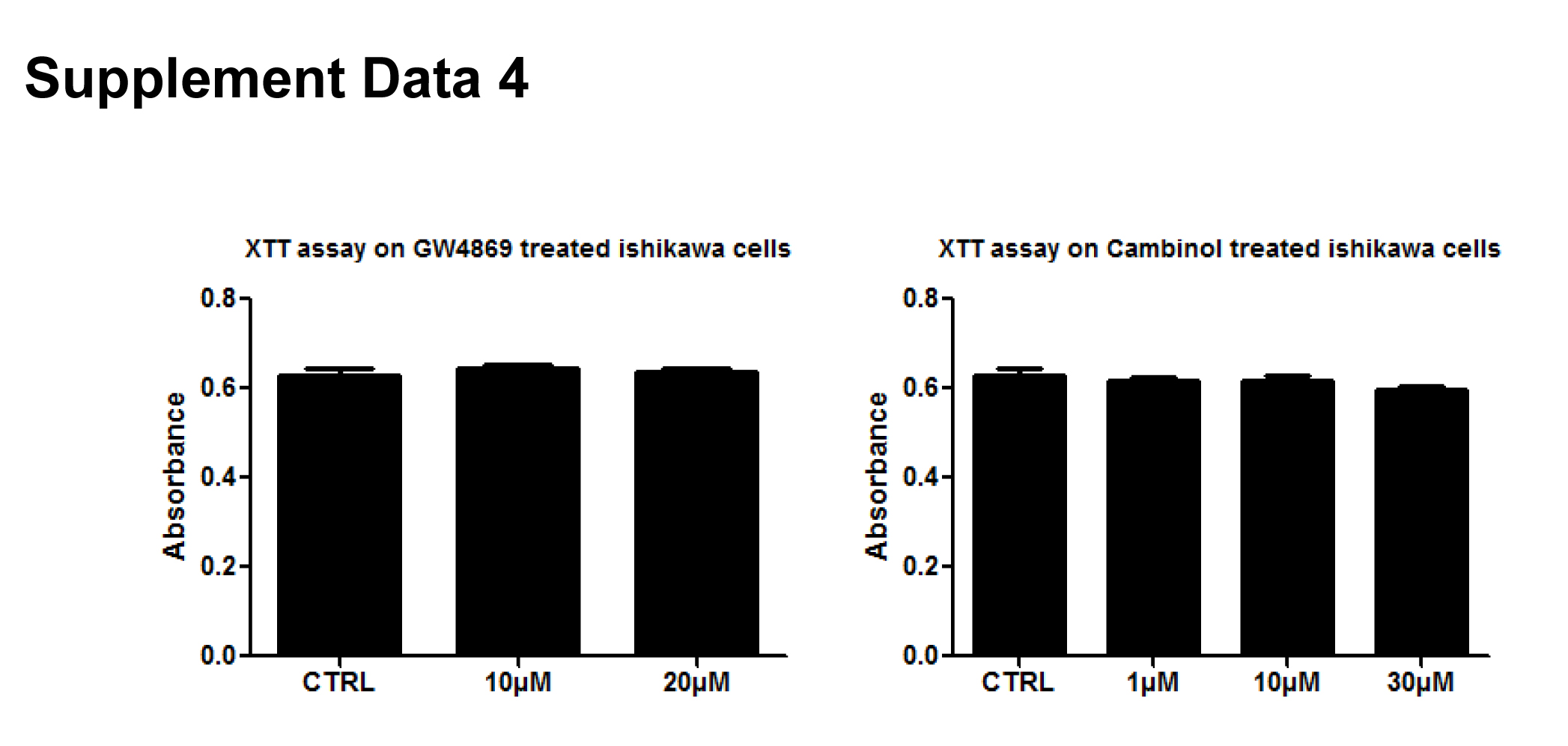

Supplement: S4 Fig — All the tested doses and the scramble control were not significantly different. (JPG) [file pone.0186534.s004.jpg]
